# Evaluation of the quality of postoperative care in neurosurgery at a national referral hospital

**DOI:** 10.3389/fsurg.2025.1665655

**Published:** 2025-11-05

**Authors:** Agbéko Komlan Doléagbénou, Ben Ousmane Djoubairou, Mazimbè Florantine Lessiou, Essossinam Kpélao, Holden Fatigba

**Affiliations:** 1 Neurosurgery Unit, CHR Lomé Commune, Lomé, Togo; 2 Faculté des Sciences de la Santé, Université de Lomé, Lomé, Togo; 3 Neurosurgery Unit, Military Hospital of Yaoundé, Faculté de Médecine de Yaoundé, Yaoundé, Cameroon; 4 Sylvanus Olympio Teaching Hospital, Lomé, Togo; 5 Faculté de Médecine, Université de Parakou, Parakou, Benin

**Keywords:** neurosurgery, postoperative care, quality assessment, surgical checklist, patient safety, Togo

## Abstract

**Background:**

The quality of postoperative care is a critical determinant of patient safety in neurosurgery, particularly in low-resource settings. This study evaluated the quality of postoperative care in the neurosurgery department of Sylvanus Olympio University Hospital in Lomé, Togo.

**Methods:**

We conducted a descriptive, cross-sectional study from October to December 2024, including patients who underwent neurosurgical procedures and received postoperative care in the neurosurgery ward. Data were collected using a structured checklist, medical record audits, and patient satisfaction surveys.

**Results:**

A total of 51 patients were included (mean age: 37.1 ± 21.6 years; male-to-female ratio: 4.67). Traumatic brain injury accounted for 45% of surgical indications. Compliance with perioperative procedural standards was observed in 64.9% of cases, while only 13.7% of postoperative prescriptions adhered to standard protocols. Postoperative complications occurring during the inpatient hospital stay were observed in 13.7% of patients, and the mortality rate was 1.96%. Despite systemic shortcomings, 64.7% of patients reported overall satisfaction with their care.

**Conclusion:**

Substantial gaps remain in the application of standardized postoperative procedures, particularly in documentation and timing of care. Strengthening written protocols, enhancing staff training, and institutionalizing regular audits may improve neurosurgical outcomes in low-resource settings.

## Introduction

The immediate postoperative period is one of the most critical phases in surgical care, especially in neurosurgery, where even minor complications can have severe neurological consequences. Ensuring safe transfer from the operating room to the recovery unit and maintaining clear, continuous communication among healthcare teams are fundamental to patient safety (1).

Evaluating the quality of postoperative care is crucial for enhancing service delivery, optimizing patient outcomes, and fostering trust in healthcare systems. Globally, the implementation of structured postoperative protocols has been shown to reduce morbidity and mortality (2). However, in low-resource settings, the quality of postoperative care often remains suboptimal due to limited infrastructure, staff shortages, and inconsistent adherence to standard procedures (3). According to the African Surgical Outcomes Study (ASOS), postoperative mortality in African countries is twice the global average, even though many patients present with lower preoperative risk profiles. Reported postoperative complication rates in sub-Saharan Africa range between 14.6% and 27.5%, highlighting significant systemic challenges (4). In neurosurgical care, the Scottish Intercollegiate Guidelines Network (SIGN) has emphasized core principles for safe postoperative management, including early identification of at-risk patients, routine neurological assessments, adequate documentation, and timely detection and management of clinical deterioration (5). However, the extent to which these standards are implemented in African neurosurgical centers remains largely undocumented.

Despite the recognized importance of standardized perioperative care, there is currently no consistent set of neurosurgical guidelines across African surgical and neurosurgical associations. Although the College of Surgeons of East, Central, and Southern Africa (COSESCA), in partnership with the AO Alliance, has recently developed guidelines for fracture management (6). Similar coordinated frameworks for neurosurgical indications remain lacking in West Africa, particularly within the West African College of Surgeons (WACS) (7). Establishing such guidelines could harmonize care delivery and improve outcomes across the region.

After a decade of neurosurgical activity at Sylvanus Olympio University Hospital in Lomé, Togo (8, 9), a structured evaluation of postoperative care practices was deemed necessary. The primary objective of this work was to assess compliance with postoperative neurosurgical care protocols, document inpatient outcomes, and identify procedural and organizational gaps at Sylvanus Olympio University Hospital. By systematically evaluating these aspects, we aim to provide evidence that informs institutional improvements and contributes to regional discussions on neurosurgical quality standards.

## Patients and methods

### Study design and setting

We conducted a descriptive, cross-sectional, and analytical study over three months, from October 1 to December 31, 2024, in the neurosurgery department of Sylvanus Olympio University Teaching Hospital (CHU Sylvanus Olympio) in Lomé, Togo. The department comprises 30 beds and is staffed by three neurosurgeons and four registered nurses, including one head nurse. The study design and reporting were guided by the Strengthening the Reporting of Observational Studies in Epidemiology (STROBE) checklist (10).

### Study population

The target population included all patients admitted to the neurosurgery department who underwent a surgical procedure and received postoperative care in the neurosurgery ward.

### Inclusion criteria

Patients were eligible for inclusion if they:

- Were admitted for a condition requiring neurosurgical intervention.
- Underwent a neurosurgical procedure during hospitalization.
- Received postoperative care in the neurosurgery ward (excluding those cared for exclusively in the ICU).
- Provided informed consent, either directly or through a legal guardian.
- Received postoperative care administered by registered nurses (excluding care provided solely by trainees).

### Exclusion criteria

Patients who underwent surgery but were not readmitted to the neurosurgery ward were excluded, as were those whose postoperative care was limited exclusively to the intensive care unit (ICU). This decision was made to ensure homogeneity in postoperative care assessment within the neurosurgery ward, recognizing that ICU-based care follows different monitoring and treatment protocols. Patients who declined to participate were also excluded from the study.

### Data collection and variables

Data were collected using a standardized checklist to evaluate compliance with postoperative protocols in both the operating room and the inpatient ward. Medical records were audited for completeness, and patients or their caregivers completed a satisfaction questionnaire covering communication, care quality, staff interaction, and hospitalization conditions.

The following variables were assessed:

- Sociodemographic data.
- Surgical details (indication, type of anesthesia, duration). Indications for surgical intervention included post-traumatic cranial pathology, degenerative spine disorders, spinal trauma, brain tumors, cerebrospinal fluid disorders, congenital malformations, and vascular lesions.
- Procedural compliance (postoperative prescriptions, timing of nursing interventions, record-keeping).
- Clinical outcomes (complications, mortality, length of stay). Complications were tracked during the inpatient stay in the neurosurgery ward. Events occurring after discharge were not systematically recorded, which represents a limitation of the study.
- Patient or caregiver satisfaction.

### Procedure evaluation

Each procedure—before anesthesia induction, before skin incision, and before the patient leaves the operating room—as well as inpatient procedures, was evaluated using a checklist. Each checklist item was assessed independently. For each evaluation step, the interviewer would mark “YES” or “NO” accordingly. The denominator was the total number of items per category.

### Definitions and evaluation criteria

- **Correct treatment administration:** defined as adherence to the physician's prescribed timing and dosage.
- **Adequate medical record: the presence of at least three of the following:** operative report, legible and detailed surgical notes, medical observations, postoperative updates, and physical examination notes.
- **Patient satisfaction:** based on responses to five domains; satisfaction required positive reactions in at least three.

### Postoperative care framework

The operating neurosurgeon provided postoperative instructions via written prescriptions. In their absence, another neurosurgeon or resident could prescribe care, provided the instructions were subsequently validated. Standardized care tools included treatment sheets, monitoring protocols, and nursing care plans.

### Ethical considerations

The Bioethics Committee approved this study for Health Research from the Togo Ministry of Health (“Comité de Bioéthique pour la Recherche en Santé (CBRS),” Ref No: 0101/2016/MS/CAB/DGS/DPLET/CBRS). Data were anonymized to ensure confidentiality.

### Statistical analysis

Data were analyzed using Epi Info version 7.5.2.0. Associations between categorical variables were assessed using the Mantel-Haenszel chi-square test, with significance set at *p* < 0.05.

## Results

### Procedure compliance

Among the 37 checklist items assessed across operating room and ward procedures, 24 items (64.9%) were found to be compliant with established protocols (Tables 1–5).

**Table 1:** Evaluation of procedures before anesthesia induction.

Criteria	Yes	No
Is there a checklist?	X	
Is there a document with essential pre-induction information?	X	
Does this document confirm		
Patient identity	X	
Surgical site	X	
Patient consent		X
Verification of anesthesia equipment and medications	X	
Oxygen saturation monitoring	X	
Allergy information	X	
Risk of difficult intubation or aspiration	X	
Hemorrhagic risk	X	
Total	**9**	**1**

**Table 2:** Evaluation of procedures before skin incision.

Criteria	Yes	No
Is there a checklist?	X	
Can the presence of team members be confirmed?		X
Anticipation of critical events		
For the surgeon		
Is there documentation of antibiotic prophylaxis?	X	
Are critical or unusual steps noted?	X	
Is the expected duration of surgery documented?	X	
Is the anticipated blood loss assessed?	X	
For the anesthesiologist:		
Is there documentation of specific patient concerns?	X	
For the nursing team		
Can instrument sterility be verified?	X	
Can equipment malfunctions or other issues be reported?	X	
Is imaging documentation available in the OR?	X	
Total	**9**	**1**

**Table 3:** Evaluation of procedures before the patient leaves the operating room.

Criteria	Yes	No
Is there a checklist?		X
Does a document verify		
Type of procedure performed?	X	
Count of instruments, gauze, and needles?	X	
Labeling of specimens?	X	
For a surgeon, an anesthesiologist, and a nurse		
Is there documentation regarding recovery and follow-up care?	X	
Total	**4**	**1**

**Table 4:** Evaluation of postoperative care procedures.

Criteria	Yes	No
Is there a checklist?		X
Is there a postoperative protocol?	X	
Is medical observation required?	X	
Is documentation of comorbidities mandatory?	X	
Is a preoperative neurological exam systematic?	X	
Is a postoperative neurological exam systematic?	X	
Is there a system for updating medical records?	X	
Is there a system for routine patient rounds?	X	
Is an operative report required?	X	
Is there a template for writing the operative report?	X	
Is a hospitalization summary required?		X
Is there a template for the hospitalization summary?		X
Total	**10**	**2**

**Table 5:** Distribution of patients based on evaluation of care staff and hospital environment.

Category	Assessment	Number	Percentage
Information about the illness	Good	50	98.04%
	Poor	1	1.96%
Quality of human interaction	Good	36	70.59%
	Poor	15	29.41%
Cost of care	Low	0	0.00%
	Medium	17	33.33%
	High	25	49.02%
	Very High	9	17.65%
Behavior of healthcare staff	Good	37	72.55%
	Poor	14	27.45%
Hospitalization conditions	Good	11	21.57%
	Poor	40	78.43%
Postoperative care	Well performed	23	45.10%
	Poorly performed	28	54.90%

### Patient demographics and clinical characteristics

**Figure 1:**
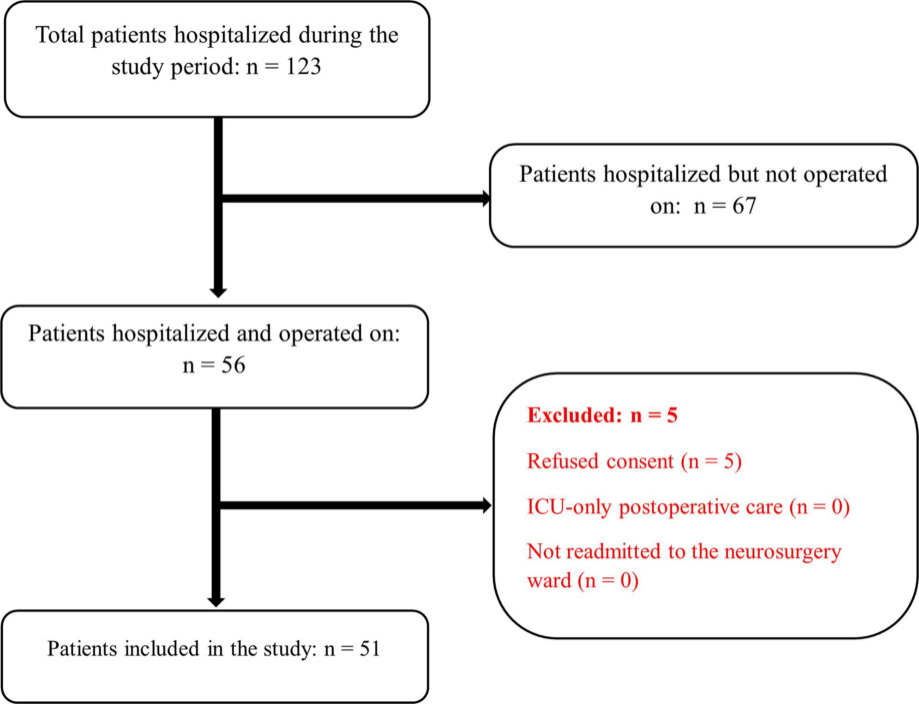
Flow diagram illustrating patient inclusion and exclusion during the study period.

A total of 51 patients were included in the study (Figure 1). There were 42 males and nine females, with a male-to-female ratio of 4.67. The mean age was 37.1 ± 21.6 years (range: 0–80 years).

The most frequent indication for surgery was post-traumatic cranial pathology (45.1%), followed by degenerative spine disorders (19.6%), spinal trauma (13.7%), brain tumors (7.8%), cerebrospinal fluid (CSF) disorders (7.8%), congenital malformations (1.9%), and vascular lesions (1.9%).

### Timing and surgical details

- The average time from admission to surgery was 7 days in 35.3% of cases, 7–14 days in 33.3%, and more than 14 days in 31.4% of cases.
- All surgical procedures were performed under general anesthesia. An anesthesiologist was physically present for 21.6% (7/51) of the surgeries.
- The average surgery duration was 3.16 ± 1.42 h (range: 1–8 h).
- No intraoperative incidents were reported.

### Postoperative care and protocol adherence

- Postoperative prescriptions deviated from the established care protocol in 86.3% of cases.
- In 76.5% of cases, the administered nursing care did not follow the prescribed timing or dosing schedule.
- The first postoperative dressing change occurred at an average of 59.3 ± 17.5 h post-surgery; in 60% of cases, it was delayed beyond 48 h.
- Nurses performed dressing changes in 82.4% of cases, neurosurgeons in 13.7%, and residents in 3.9%.
- The average length of hospital stay was 12.6 ± 9.4 days (range: 2–36 days).

Outcomes were favorable in 86.28% of patients. Complications occurred in 7 patients (13.72%): 2 surgical site infections (3.92%), three pulmonary infections (5.88%), 1 case of meningitis (1.96%), and 1 case of a sacral pressure ulcer (1.96%). One patient (1.96%) died postoperatively.

### Postoperative outcomes

- Clinical outcomes were favorable in 86.3% of patients.
- Postoperative complications were observed in 7 patients (13.7%), including:
  ○ Surgical site infections (3.9%)
  ○ Pulmonary infections (5.9%)
  ○ Meningitis (1.9%)
  ○ Sacral pressure ulcers (1.9%)
  ○ Venous thromboembolism (deep vein thrombosis or pulmonary embolism) was not systematically tracked.
- One patient (1.96%) died during the postoperative period.

### Medical record assessment

Medical records were judged adequately maintained in 52.9% of cases. However:

- Postoperative updates were missing in 72.6% of files.
- Operative reports were absent in 17.7% of cases.
- No hospitalization summary was found in any patient record.

### Patient satisfaction

- Overall satisfaction was reported by 64.7% of patients.
- However, 78.4% rated hospitalization conditions as poor, and 54.9% reported dissatisfaction with postoperative care (Table 5).
- High subjective satisfaction was primarily linked to interpersonal aspects, including caregiver behavior and communication, rather than technical or environmental factors.

## Discussion

This study evaluated postoperative care practices in a neurosurgical unit within a resource-constrained tertiary hospital in Togo. The findings highlight both systemic strengths and significant gaps in the implementation of standard care protocols. This is the first study of its kind in Togo and one of the few in sub-Saharan Africa to systematically assess postoperative neurosurgical care using checklists, patient feedback, and procedural audits.

### Patient management, compliance with protocols and documentation

Neurotraumatology accounts for a significant portion of neurosurgical activity in our setting (6, 8–10). In our study, traumatic brain injuries were the most common indication for surgery (33.33%), followed by degenerative spinal diseases (19.61%) and spinal trauma (13.73%). Our findings are similar to those reported in similar conditions (8, 11–15).

Although checklists were implemented in both the operating room and the ward, procedural compliance was suboptimal (64.9%). This suggests that standardized protocols have not yet been fully integrated into routine clinical practice. In high-income settings, the adoption of checklists has been associated with a reduction in morbidity and an improvement in safety for neurosurgical patients (1). Our findings align with other African studies, which have shown inconsistent adherence to safety measures due to staff shortages, insufficient training, and inadequate institutional reinforcement of protocols (3, 16).

Medical documentation was particularly weak. Only 52.9% of patient files were adequately maintained, with missing operative reports and absent discharge summaries in all cases. Such deficiencies compromise continuity of care and quality assessment and may reflect high workload, inadequate supervision, or insufficient institutional accountability.

### Quality of nursing and postoperative care

One of the most striking findings was the poor adherence to postoperative prescriptions. In 86.3% of cases, prescriptions deviated from standard protocols, and 76.5% of administered care did not match physician orders. These results indicate serious gaps in care coordination and clinical governance. Similar trends have been reported in other African surgical studies, where deviations from best practices often arise from overburdened nurses and a lack of standardized workflows (4, 16).

Delayed dressing changes (occurring beyond 48 h in 60% of cases) and inconsistent postoperative neurological assessments further highlight the fragility of postoperative protocols in our setting.

The administration of treatments, wound care, and the prevention of complications requires the harmonization of practices. Better coordination among care teams and greater adherence to existing protocols are crucial for improving care quality and patient safety.

### Postoperative complications and mortality

The postoperative complication rate in our cohort was 13.7%, which aligns with other studies from sub-Saharan Africa that report rates between 10% and 28% [4, 16. The relatively young mean age of patients (37 years) may have contributed to a lower-than-expected mortality rate (1.96%), which compares favorably with the 8.9% reported in other regional studies (11). Mortality rates vary by type of surgery. In one English study, the 30-day mortality rate after scheduled neurosurgical procedures ranged from 0 to 0.7%, except for neuro-oncology, where it was 2.3%. For emergency neurosurgical procedures, the rate varied between 0% and 11% (17).

Longer operative times (>3 h on average) and suboptimal postoperative monitoring are recognized risk factors for complications such as surgical site and pulmonary infections, both of which were observed in this study (18). Our findings align with those of the ASOS (African Surgical Outcomes Study), which found that lack of trained personnel and low levels of supervision contribute to the occurrence of postoperative complications (16).

### Patient satisfaction

Despite systemic deficiencies, 64.7% of participants reported overall satisfaction with the patient experience. However, this contrasted with the 78.4% who judged hospitalization conditions to be poor and 54.9% who were dissatisfied with postoperative care. These findings suggest that interpersonal aspects—such as staff behavior and communication—may mitigate negative perceptions of technical or logistical shortcomings. Nonetheless, subjective satisfaction should not mask the objective quality gaps in care delivery.

### Implications for practice

An additional consideration concerns the withdrawal of life-sustaining treatment (WLST) in postoperative neurosurgical patients. Although we did not directly capture WLST data, recent studies (19) demonstrate that neurosurgeons' decisions are strongly influenced by patient age, Glasgow Coma Scale score, pupillary response, comorbidities, and the potential for long-term vegetative state. While our study did not directly address these issues, integrating them into future evaluations would broaden the scope of postoperative care quality assessments and strengthen their ethical dimension.

Our results underline the urgent need for structured quality improvement initiatives. Beyond institutional improvement, this evaluation also highlights the potential for regional impact. Structured assessments of postoperative care, such as ours, can inform broader efforts to establish standardized neurosurgical guidelines in conjunction with professional bodies, including WACS and COSESCA.

### Strengths and limitations

This study was conducted in a single center with a modest sample size (51 patients over three months), which limits the generalizability of the findings. Future research involving multicenter and longitudinal designs is necessary to strengthen external validity.

This study has several limitations. First, it was conducted in a single center, which restricts the generalizability of the findings to other institutions in Togo or the wider region. Second, the relatively small sample size limits the statistical power of our analyses. Third, observer bias may have influenced the checklist evaluations, despite efforts to standardize data collection. Fourth, patient satisfaction responses may have been subject to courtesy bias, particularly given the cultural context. Finally, complications were tracked only during inpatient hospitalization in the neurosurgery ward; events occurring after discharge were not systematically captured, which may underestimate the actual burden of postoperative morbidity.

## Conclusion

This study presents a critical snapshot of postoperative neurosurgical care in a tertiary hospital in Togo, highlighting significant procedural and organizational deficiencies despite the presence of written protocols. While overall patient outcomes and satisfaction were relatively acceptable, major issues were identified in care coordination, nursing execution, and medical documentation.

To improve patient safety and the quality of neurosurgical care in resource-limited settings, urgent action is needed in three key areas:

1. ***Reinforcement of protocol adherence*** through institutional commitment and clinical supervision.
2. ***Capacity building*** *via* ongoing staff training focused on perioperative and postoperative standards.
3. ***Implementation of quality assurance systems***, including regular audits and feedback mechanisms.

These measures are not only feasible but also essential for strengthening surgical systems in low-income countries, where even minor improvements can yield significant gains in outcomes.

## Data Availability

The raw data supporting the conclusions of this article will be made available by the authors, without undue reservation.
